# Effect of Interactive eHealth Interventions on Improving Medication Adherence in Adults With Long-Term Medication: Systematic Review

**DOI:** 10.2196/18901

**Published:** 2021-01-08

**Authors:** Bart P H Pouls, Johanna E Vriezekolk, Charlotte L Bekker, Annemiek J Linn, Hein A W van Onzenoort, Marcia Vervloet, Sandra van Dulmen, Bart J F van den Bemt

**Affiliations:** 1 Department of Rheumatology Research Sint Maartenskliniek Nijmegen Netherlands; 2 Department of Pharmacy Radboud Institute for Health Sciences Radboud University Medical Centre Nijmegen Netherlands; 3 Amsterdam School of Communication Research University of Amsterdam, Amsterdam Netherlands; 4 Department of Clinical Pharmacy Amphia Hospital Breda Netherlands; 5 Department of Clinical Pharmacy and Toxicology Maastricht University Medical Centre Maastricht Netherlands; 6 Nivel (Netherlands Institute for Health Services Research) Utrecht Netherlands; 7 Department of Primary and Community Care Radboud Institute for Health Sciences Radboud University Medical Centre Nijmegen Netherlands; 8 Faculty of Health and Social Sciences University of South-Eastern Norway Drammen Norway

**Keywords:** eHealth, mHealth, medication adherence, interventions, long-term conditions

## Abstract

**Background:**

Medication nonadherence leads to suboptimal treatment outcomes, making it a major priority in health care. eHealth provides an opportunity to offer medication adherence interventions with minimal effort from health care providers whose time and resources are limited.

**Objective:**

The aim of this systematic review is twofold: (1) to evaluate effectiveness of recently developed and tested interactive eHealth (including mHealth) interventions on medication adherence in adult patients using long-term medication and (2) to describe strategies among effective interventions.

**Methods:**

MEDLINE, EMBASE, Cochrane Library, PsycINFO, and Web of Science were systematically searched from January 2014 to July 2019 as well as reference lists and citations of included articles. Eligible studies fulfilled the following inclusion criteria: (1) randomized controlled trial with a usual care control group; (2) a total sample size of at least 50 adult patients using long-term medication; (3) applying an interactive eHealth intervention aimed at the patient or patient’s caregiver; and (4) medication adherence as primary outcome. Methodologic quality was assessed using the Cochrane risk of bias tool. Selection and quality assessment of studies were performed by 2 researchers (BP and BvdB or JV) independently. A best evidence synthesis was performed according to the Cochrane Back Review Group.

**Results:**

Of the 9047 records screened, 22 randomized clinical trials were included reporting on 29 interventions. Most (21/29, 72%) interventions specified using a (mobile) phone for calling, SMS text messaging, or mobile apps. A majority of all interactive interventions (17/29) had a statistically significant effect on medication adherence (*P*<.05). Of these interventions, 9 had at least a small effect size (Cohen d ≥ 0.2) and 3 showed strong odds for becoming adherent in the intervention group (odds ratio > 2.0). Our best evidence synthesis provided strong evidence for a positive effect of interventions using SMS text messages or interactive voice response, mobile app, and calls as mode of providing adherence tele-feedback. Intervention strategies “to teach medication management skills,” “to improve health care quality by coordinating medication adherence care between professionals,” and “to facilitate communication or decision making between patients and health care providers” also showed strong evidence for a positive effect.

**Conclusions:**

Overall, this review supports the hypothesis that interactive eHealth interventions can be effective in improving medication adherence. Intervention strategies that improve patients’ treatment involvement and their medication management skills are most promising and should be considered for implementation in practice.

## Introduction

Long-term medication aims to reduce the risk of disease progression, comorbidity, and mortality [1]. These outcomes will only be reached when patients adhere to their medication. Presumably 50% of all patients with long-term medication are nonadherent, leading to suboptimal treatment outcomes [2,3]. Although improvement on clinical outcomes is the ultimate treatment goal, measuring adherence to long-term medication allows for comparison across chronic conditions.

Medication adherence is defined as the extent to which medication taking behavior corresponds with the medication regimen agreed upon with the health care professional [4]. Medication-taking behavior is influenced by different factors such as experience, beliefs, and culture, making it multifaceted. Moreover, medication-taking behavior can differ between each drug and may change over time. Targeted, timely interventions enhancing medication adherence have therefore become one of the major priorities in health care. Despite efforts, randomized controlled trials have demonstrated limited effectiveness of medication-enhancing interventions [5-9]. Besides, effective interventions differed markedly and did not apply similar intervention strategies, making comparisons or meta-analysis difficult [3,5-7].

eHealth might provide an opportunity to offer accessible, interactive, timely, and feasible medication adherence interventions that require minimal effort from health care providers whose time and resources are limited. eHealth or telemedicine—these words are used interchangeably—is defined as the use of information and communication technology in health care [10]. These technologies can facilitate tailored and interactive solutions such as targeted education, consistent support, and skill acquisition. Thus, the multifaceted and versatile medication-taking behavior can well be targeted by eHealth interventions.

eHealth seems a promising way forward but recent systematic reviews showed conflicting results for eHealth interventions on improving medication adherence [11-14]. These reviews focused on single long-term conditions and have led to fragmented knowledge on the effectiveness and strategies of eHealth interventions. Evidence on eHealth interventions should be clustered to comprehensively investigate effectiveness of eHealth interventions and facilitate generalizability of study findings. Sieben et al [5] and Linn et al [15] found promising results across long-term conditions but the fast developments in eHealth render those results outdated, their definition of eHealth as “internet” was too narrow, and included studies had methodological limitations. Therefore the aim of our systematic review is twofold: (1) to evaluate effectiveness of recent interactive eHealth interventions on medication adherence in adult patients using long-term medication and (2) to describe applied strategies within effective interventions.

## Methods

This systematic review adheres to the PRISMA (Preferred Reporting Items for Systematic Reviews and Meta-analyses) statement [16] and was completed according to the registered protocol PROSPERO 2019 CRD42019088873 [17].

### Search Strategy and Study Selection

Searches were undertaken in MEDLINE, EMBASE, Cochrane Library, PsycINFO, and Web of Science to identify eligible studies. The search strategy comprised 3 blocks: eHealth, medication adherence, and randomized clinical trial (see Multimedia Appendix 1 for the MEDLINE search). Reference lists and citations of included studies were checked to ensure literature saturation. Titles and abstracts were screened and full-text articles were assessed by 2 researchers (BP and BvdB or BP and JV) independently based on the inclusion criteria below. Discrepancies between researchers were resolved through discussion or by reaching consensus with the third researcher (BvdB or JV).

### Eligibility Criteria

Eligible studies fulfilled the following inclusion criteria: (1) randomized controlled trial with a usual care control group; (2) applying an interactive eHealth intervention aimed at the patient or patient’s caregiver; (3) medication adherence as primary outcome; (4) a total sample size of at least 50 adult patients using long-term medication as determined by Zwikker et al [18]; and (5) published between 2014 and July 2019. Only peer-reviewed English full-text articles were included. We considered all interventions solely applied over distance as eHealth interventions (eg, online portals, telephone calls). Blended care interventions, where face-to-face contact is combined with online components, were excluded. Interventions were considered interactive if there was tele-feedback regardless by whom on medication adherence (eg, bidirectional text messaging, sending adherence reports). Only validated medication adherence outcomes (ie, objective measures or subjective measures that have been compared to objective measures) were taken into account.

### Quality of Evidence

Two researchers (BP and JV) independently assessed the internal validity of included studies using the Cochrane Collaboration’s tool for assessing risk of bias [19]. Seven domains were scored as having low (+), high (–), or unclear (?) risk of bias. Because blinding of participants and personnel is hardly feasible in studies evaluating interventions aimed at adherence, this domain was considered high risk (–) for all studies. Studies with a positive score (+) on at least five domains were considered high-quality studies. If relevant information was not reported, the corresponding author was contacted to request additional information. When no additional relevant information was provided, the risk of bias domain was scored as unclear (?).

### Data Extraction

A standardized template was made to extract data on study characteristics, eHealth interventions, and medication adherence outcomes. Details of the eHealth interventions were extracted according to the Template for Intervention Description and Replication (TIDieR) checklist [20]. Additionally, the mode of adherence tele-feedback of each eHealth intervention was extracted. We distinguished the following modes of adherence tele-feedback: monitoring device, SMS text messaging, interactive voice response (IVR), mobile app, call, or e-training. Intervention strategies were categorized based on the strategies defined by Lowe et al [21] to support behavior change (ie, strategies focusing on adopting treatment behaviors); to inform and educate; to support (ie, strategies assisting consumers with their medicines use such as peer support); to teach skills; to minimize risk and harms (ie, strategies focusing on preventing or managing adverse events); to facilitate communication or decision making; and to improve health care quality (ie, strategies improving, coordinating, or integrating the delivered care). Only the adherence outcome at study endpoint was extracted where magnitude of the intervention effect would be most apparent. For continuous outcomes Cohen *d* for assessing effect size was calculated if means and SDs were provided [22]. Dichotomous outcomes were recalculated to odds ratio (OR) where OR ≥2.0 is deemed to be a strong OR [23]. Additionally, if the authors reported a statistical significance favoring the intervention group compared to the control group, this was scored positive (+). A negative score (–) means there was no statistically significant difference to report. Data were extracted by one researcher (BP) and checked for accuracy by a second researcher (JV).

### Data Analysis

Statistical data pooling was not feasible due to heterogeneity between studies and interventions. Therefore a best evidence synthesis was performed to examine the effectiveness of interactive eHealth interventions on medication adherence. The Cochrane Back Review Group defines 4 levels of evidence: strong, moderate, limited, and conflicting evidence [24]. Strong evidence reflects consistent (ie, 75% or more of the studies report results in the same direction) results among 2 or more high quality studies. Moderate evidence reflects consistent results of 1 high-quality study and 2 or more lower-quality studies. Limited evidence reflects the result of 1 lower-quality study. Conflicting evidence reflects inconsistent results among 2 or more studies. If there were 2 or more high-quality studies, the lower-quality studies were disregarded in the best evidence synthesis. A post-hoc sensitivity analysis was performed to examine the robustness of the best evidence synthesis using a more stringent cut-off score (6 out of 7 instead of 5 out of 7 domains graded as low risk of bias) for determining the quality of the studies.

## Results

### Search Results

**Figure 1:**
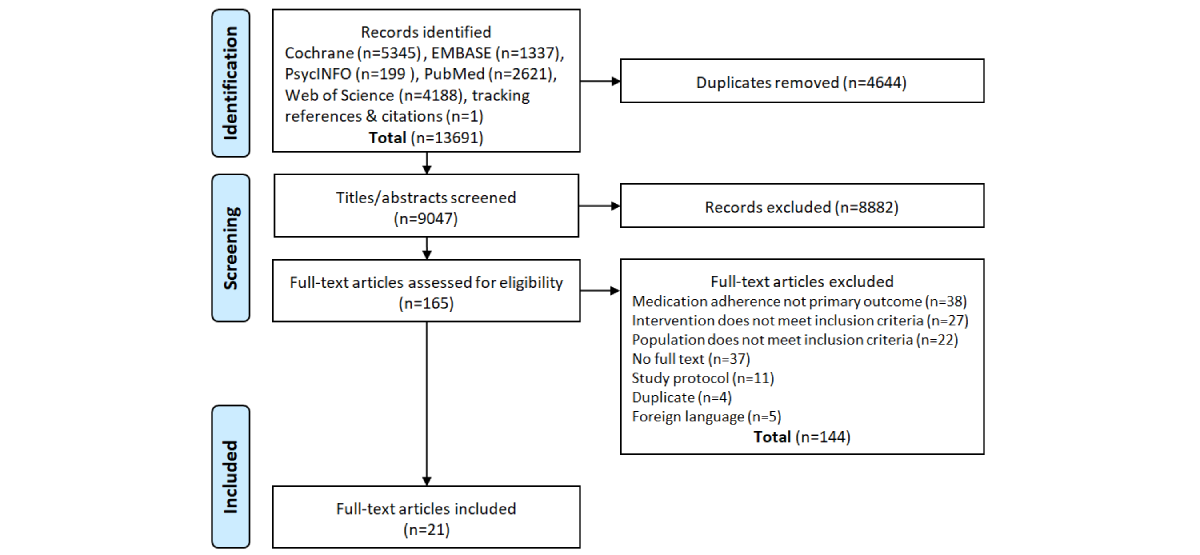
PRISMA flow diagram of study search and selection.

Figure 1 shows a flow diagram of the literature search which provided a total of 9047 publications for screening and yielded 21 articles reporting on 29 interactive eHealth interventions [25-45]. One article, by Reese et al [34], reported on 2 studies. Five studies reported on more than 1 intervention [28,34,35,38,45].

### Risk of Bias Assessment

**Figure 2:**
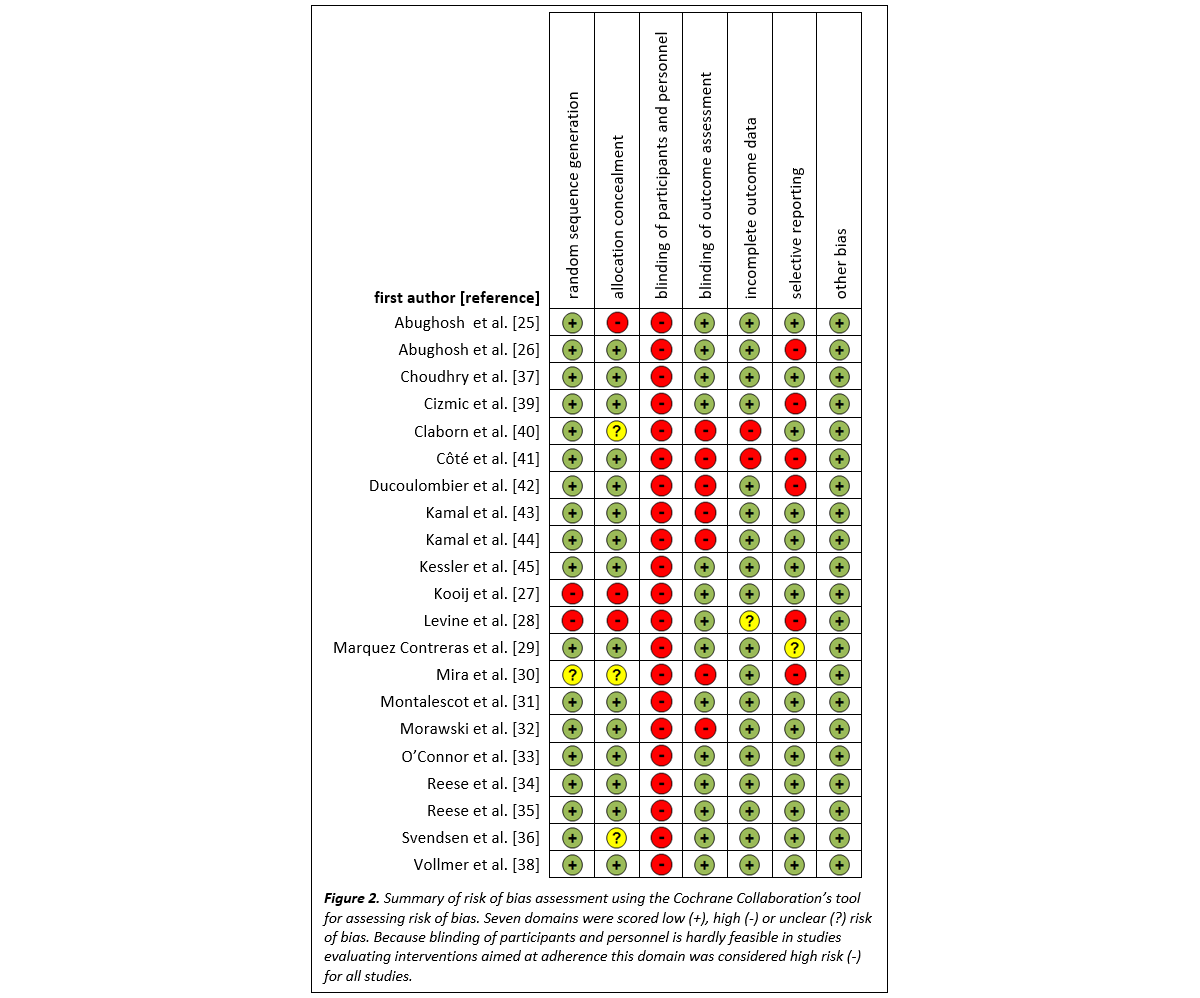
Summary of risk of bias assessment using the Cochrane Collaboration’s tool for assessing risk of bias.

Fifteen studies had a positive score on at least five domains and were regarded high-quality studies as shown in Figure 2. Two studies had the lowest score with 2 out of 7 domains scored as positive.

### Description of Study Population

Over half of the studies (13/22) included long-term medication for cardiovascular disease, diabetes, or both. Seven studies focused on other, single long-term conditions, leaving 2 studies that looked at any long-term conditions where long-term medication was in use.

The smallest study reported on 70 participants at baseline and the largest study involved 21,752 participants. Because all studies were randomized, baseline characteristics of the different groups were generally the same.

### Description of Study Design

Follow-up was short (ie, less than 6 months) in 11 studies and long (at least six months) in 11 studies. The primary medication adherence outcome of each of the studies was mainly assessed objectively using medication monitoring devices, pharmacy prescription data, and serum levels. The remaining 6 studies measured adherence subjectively with validated self-report questionnaires (eg, Immunosuppressant Therapy Adherence Instrument).

### Description of eHealth Interventions and Intervention Strategies

Twenty-nine different interactive eHealth interventions were evaluated as shown in Table 1. Most (21/29, 72%) interventions specified using a (mobile) phone for calling, SMS text messaging, or mobile apps.

Most (25/29) interventions were aimed at the patient, 3 interventions were aimed at the caregiver, and another was aimed at either patient or caregiver.

Sixteen interventions were provided through automated software without involvement of a health care professional: 6 mobile apps, 5 monitoring devices, 3 SMS text messages or IVR interventions, and 2 e-training modules through an online portal. Another 7 interventions were provided through automated software in combination with tele-feedback by a health care professional or caregiver: 4 monitoring devices, 2 IVR or SMS text message interventions, 1 e-training. The 6 remaining interventions were telephone calls performed by health care professionals.

Regarding intervention strategies, nearly all (23/29, 79%) interventions aimed at informing and educating patients and just over half (15/29, 52%) sought to support patients by providing assistance and encouragement. All other strategies (eg, teaching skills, facilitating communication or decision making) were less frequently applied (see Multimedia Appendix 2).

### Effectiveness of eHealth Interventions on Medication Adherence

Overall, 17 interventions yielded a statistically significant improvement of medication adherence compared to the control group (Table 2). For 14 of these interventions an effect size (Cohen *d*) could also be calculated; 2 interventions reported a large effect size (Cohen *d* ≥ 0.8) [25,29], 4 had a medium effect size (Cohen *d* ≥ 0.5 < 0.8) [35,43,45], 3 had a small effect size (Cohen *d* ≥ 0.2 < 0.5) [26,32,35], and 5 interventions had a negligible effect size (Cohen *d* < 0.2) [27,30,37,38]. For the remaining 3 interventions an OR could be calculated which showed strong odds for becoming adherent in the intervention group (OR ≥ 2.0) [36,39,42].

**Table 1:** Characteristics of the eHealth interventions.

Study and medication		Adherence inclusion criterion	Intervention arm (n)	Control arm (n)	Follow-up (in weeks)	Mode of adherence tele-feedback	Description of the intervention
**Levine et al [28]**							
	Tacrolimus	None	38	50	13	App	Transplant Hero is an interactive alarm to remind patients to take their medications as well as providing educational content.
	Tacrolimus	None	20	50	13	App and smart-watch	Transplant Hero (see above) combined with a smartwatch that displayed the reminder notifications.
**Cizmic et al [39]**							
	Bisphosphonates	None	127	118	4	IVR^a^	An IVR call focusing on known reasons for not initiating therapy. If the medication was not picked up 7 days after receiving the call, a reminder letter was sent.
**O’Connor** **et al [33]**							
	Antihypertensives or medication for lowering blood glucose or cholesterol	None	1220	1158	9	Call	A single-protocol-structured telephone call from an interventionist using positive reinforcement and probing for reasons of nonadherence.
**Kessler et al [45]**							
	Statins	<80%	51	34	26	Device	A wireless pill bottle generated an alert message, sent to the participant, if medication was missed the previous day and at least once in the 2 prior days.
	Statins	<80%	46	34	26	Device	A wireless pill bottle generated an automated alert message (see above), sent to the participant and a designated caregiver.
**Márquez Contreras** **et al [29]**							
	Antihypertensives	None	73	75	52	App	The AlerHTA app aimed to promote health education in hypertension and remind for both appointments and medication intake time.
**Montalescot** **et al [31]**							
	Apixaban	None	579	583	24	e-Training	An education program consisting of an education booklet, one or more reminder tools chosen by the participant, and access to a telephone clinic.
**Reese et al [34]**							
	Statins	<80%	67	67	13	Device	PROMOTE-1: a wireless pill bottle generated a weekly adherence report in which the patient’s adherence was compared to other patients.
	Statins	<80%	67	67	13	Device	PROMOTE-2: a wireless pill bottle generated a weekly adherence report.
	Statins	<80%	50	50	13	Device	SUPPORT-1: a wireless pill bottle generated a daily adherence report.
	Statins	<80%	50	50	13	Device	SUPPORT-2: a wireless pill bottle generated a weekly adherence report.
	Statins	<80%	50	50	13	Device	SUPPORT-3: a wireless pill bottle generated an email alert if the patient missed a dose the previous day.
**Reese et al [35]**							
	Tacrolimus	None	40	40	26	Device	A wireless pill bottle generated an alert when medication was due and patients could select additional reminders such as SMS text messages, calls, or emails.
	Tacrolimus	None	40	40	26	Device	A wireless pill bottle generated an alert (see above). If adherence decreased to <90% in a 14-day period, the study coordinator would call the patient and notify the involved HCPs^c^.
**Svendsen et al [36]**							
	Calcipotriol/betamethasone foam	none	68	66	4	App	An app which provided once-daily reminders and information on number of treatment applications and amount of prescribed foam applied.
**Abughosh et al [25]**							
	RAS^b^ inhibitors	<80%	87	99	26	Call	A brief telephone intervention by pharmacists to remind the patients of their overdue refill and to identify potential adherence barriers.
**Abughosh et al [26]**							
	RAS inhibitors	<80%	248	495	26	Call	Six motivational interviewing phone calls by pharmacy students to identify potential adherence barriers and provide guidance to address these barriers.
**Choudhry et al [37]**							
	Antihypertensives or medication for lowering blood glucose or cholesterol	<80%	2038	2040	52	Call	Tailored telephone consultation to develop a shared plan to improve adherence and disease control. At 6 and 9 months progress reports were mailed.
**Ducoulombier et al [42]**							
	Bisphosphonates and strontium ranelate	None	79	85	52	Call	Bimonthly telephone follow-up to motivate patients to maintain good adherence, detect difficulties in compliance, and recall the importance of treatment continuation.
**Kooij et al [27]**							
	Bisphosphonates, RAS inhibitors, and statins	None	2008	2914	52	Call	Telephone counselling 7-21 days after the start of therapy assessing practical and perceptual barriers and providing information and motivation.
**Vollmer et al [38]**							
	RAS inhibitors and statins	<90%	7247	7255	52	IVR	An IVR call when (over)due for a refill providing patient education and refill support.
	RAS inhibitors and statins	<90%	7250	7255	52	IVR	In addition to IVR calls (see above), a reminder letter was sent if they were 60-89 days overdue, a call was made if they were ≥90 days overdue, and primary care provider informed. Patients also received a personalized health report, a pill organizer, and bimonthly mailings.
**Claborn et al [40]**							
	Highly active antiretroviral therapy	<95%	47	50	4	e-Training	eLifeSteps: a single-session, self-paced multimedia intervention tackling practical and psychological adherence barriers accompanied with a workbook.
**Côté** **et al [41]**							
	Immunosuppressants	None	35	35	26	e-Training	Transplant-TAVIE was composed of 3 interactive Web-based sessions by a virtual nurse aimed at developing and reinforcing self-management skills required for medication intake.
**Kamal et al [43]**							
	Preventive medication for stroke	None	100	100	8	SMS text messages	SMS4stroke sent automated customized SMS text message reminders to either patient or caregiver.
**Kamal et al [44]**							
	Statins and antiplatelets	None	99	98	13	IVR and SMS	Daily IVR call services, daily prescription-tailored medication reminders, and once weekly life style modification messages.
**Mira et al [30]**							
	All medication allowed, >2	None	51	49	13	App	A tablet-based medication self-management app (ALICE) with medication reminders and medication information such as pictures, interactions, storage instructions, and common errors in medication use.
**Morawski et al [32]**							
	Antihypertensives	None	209	202	12	App	The MediSafe app is a medication reminder app with additional functions such as adherence reports, tracking of measurements, and peer support.

^a^IVR: interactive voice response.

^b^RAS: renin–angiotensin system.

^c^HCP: health care professional.

**Table 2:** Adherence measure and medication adherence results of the studies reviewed.

Adherence measure, study, and results on medication adherence			Statistically significant^a^
**Serum level (0** **-** **100)**			
	**Levine et al [28]**		
		The coefficient of variability (SD/mean × 100) of tacrolimus levels was 33.0 for the intervention group and 32.8 for the control group (Cohen *d* = 0.01).	–
		The coefficient of variability was 33.8 for the intervention group and 32.8 for the control group (Cohen *d* = 0.07).	–
**Fill first prescription (0** **%-** **100%)**			
	**Cizmic et al [39]**		
		49% of the intervention group filled their first prescription compared to 31% of the control group (OR^b^ 2.17; 95% CI 1.29-3.67).	+
	**O’Connor** **et al [33]**		–
		84% of the intervention group filled their first prescription compared to 84% of the control group (OR 0.94; 95% CI 0.79-1.11).	–
**Bottle openings (0%-100%)**			
	**Kessler et al [45]**		
		Average daily adherence was 53% for the intervention group and 36% for the control group (Cohen *d* = 0.70).	+
		Average daily adherence was 55% for the intervention group and 36% for the control group (Cohen *d* = 0.70).	+
	**Márquez Contreras** **et al [29]**		
		Average daily adherence was 86% for the intervention group and 63% for the control group (Cohen *d* = 4.72).	+
	**Montalescot** **et al [31]**		
		Average daily adherence was 92% for the intervention group and 92% for the control group (Cohen *d* = 0.02).	–
	**Reese et al [34]**		
		Average daily adherence was 77% for the intervention group and 75% for the control group.	–
		Average daily adherence was 71% for the intervention group and 75% for the control group.	–
	**Reese et al [34]**		
		Average daily adherence was 73% for the intervention group and 79% for the control group.	–
		Average daily adherence was 75% for the intervention group and 79% for the control group.	–
		Average daily adherence was 75% for the intervention group and 79% for the control group.	–
	**Reese et al [35]**		
		Average daily adherence (during the final 90 days) was 78% for the intervention group and 55% for the control group (Cohen *d* = 0.37).	+
		Average daily adherence (during final 90 days) was 88% for the intervention group and 55% for the control group (Cohen *d* = 0.57).	+
	**Svendsen et al [36]**		
		66% of the intervention group was considered adherent compared to 38% of the control group (OR 3.22; 95% CI 1.53-6.80).	+
**PDC^c,d ^** **(0%** **-** **100%)**			
	**Abughosh et al [25]**		
		PDC was 58% for the intervention group and 29% for the control group (Cohen *d* = 1.32).	+
	**Abughosh et al [26]**		
		PDC was 66% for the intervention group and 57% for the control group (Cohen *d* = 0.26).	+
	**Choudhry et al [37]**		
		PDC was 46% for the intervention group and 42% for the control group (Cohen *d* = 0.12).	+
	**Ducoulombier et al [42]**		+
		65% of the intervention group was considered adherent compared to 33% of the control group (OR 3.71; 95% CI 1.94-7.07).	+
	**Kooij et al [27]**		
		PDC was 81% for the intervention group and 76% for the control group (Cohen *d* = 1.34).	+
	**Vollmer et al [38]**		
		PDC was 58% for the intervention group and 56% for the control group (Cohen *d* = 2.09).	+
		PDC was 59% for the intervention group and 56% for the control group (Cohen *d* = 2.14).	+
**AACTGAI^e ^** **(0%** **-** **100%)**			
	**Claborn et al [40]**		
		Adherence was 81% for the intervention group and 81% for the control group (Cohen *d* = –0.03).	–
**ITAS^f^** **(0-12)**			
	**Côté** **et al [41]**		
		Mean ITAS score was 11.7 in the intervention group and 11.3 in the control group (Cohen *d* = 0.30).	–
**MMAS^ g ^** **(0-8)**			
	**Kamal et al [43]**		
		Mean MMAS score was 7.4 in the intervention group and 6.7 in the control group (Cohen *d* = 0.62).	+
**MMAS (0-8)**			
	**Kamal et al [44]**		
		Mean MMAS score was 7.3 in the intervention group and 7.1 in the control group (Cohen *d* = 0.03).	–
**MMAS-4 (0-8)**			
	**Mira et al [30]**		
		Mean MMAS score was 7.4 in the intervention group and 7.3 in the control group (Cohen *d* = 0.12; not corrected for baseline).	+
**MMAS (0-8)**			
	**Morawski et al [32]**		
		Mean MMAS score was 6.3 in the intervention group and 5.7 in the control group (Cohen *d* = 0.35).	+

^a^As reported by the authors. + indicates *P*<.05 favoring intervention and – indicates *P*>.05 (no significant difference between groups).

^b^OR: odds ratio.

^c^PDC: percentage of days covered.

^d^All PDC outcomes were based on refill data; pill counts were considered separately.

^e^AACTGAI: Adult AIDS Clinical Trials Group Adherence Instrument.

^f^ITAS: Immunosuppressant Therapy Adherence Instrument.

^g^MMAS: Morisky Medication Adherence Scale.

Further details of the study, population, intervention, and outcomes can be found in the extraction database provided as Multimedia Appendix 5.

The best evidence synthesis (Table 3) showed strong evidence for a positive effect for SMS text messages or IVR, mobile apps, and calls as mode of adherence tele-feedback. The evidence for e-training was weak and for monitoring devices conflicting.

In the post hoc sensitivity analysis the criteria for a high-quality study were more stringent (6 out of 7 domains graded as low risk of bias). The sensitivity analysis showed that the strong evidence for a positive effect for SMS or IVR as mode of adherence tele-feedback remained, whereas the evidence turned to conflicting for interventions delivered through mobile apps and calls (see Multimedia Appendix 3).

The level of evidence of the intervention strategies was also assessed. There was strong evidence for a positive effect of strategies to teach skills, to facilitate communication or decision making, and to improve health care quality. For all other intervention strategies (eg, to support, to inform and educate) there was conflicting evidence (see Multimedia Appendix 4).

**Table 3:** Results of the best evidence synthesis.

Mode of adherence tele-feedback and quality		Statistically significant^a^	Level of evidence
Monitoring device			Conflicting evidence
	9 HQ^b^ interventions	+, +, +, +, –, –, –, –, –	
	0 LQ^c^ interventions		
SMS text messaging or IVR^d^			Strong evidence for a positive effect
	5 HQ interventions	+, +, +, +, –	
	0 LQ interventions		
Mobile app			Strong evidence for a positive effect
	3 HQ interventions	+, +, +	
	3 LQ interventions	+, –, –	
Call			Strong evidence for a positive effect
	4 HQ interventions	+, +, +, –	
	2 LQ interventions	+, +	
e-Training			Moderate evidence for no effect
	1 HQ intervention	–	
	2 LQ interventions	–, –	

^a^+ indicates *P*<.05 favoring intervention; – indicates *P*>.05 (no significant difference between groups). In grading the level of evidence low-quality studies were disregarded when there were 2 or more high-quality studies.

^b^HQ: high quality.

^c^LQ: lower quality.

^d^IVR: interactive voice response.

## Discussion

### Principal Findings

This systematic review examined the effectiveness of interactive eHealth interventions to improve medication adherence in patients using long-term medication published between 2014 and 2019. A majority, 17/29 interactive interventions, had a statistically significant (*P*<.05) effect on medication adherence. There was strong evidence for a positive effect for interventions using SMS or IVR, mobile apps, and calls as mode of adherence tele-feedback. Intervention strategies to teach skills, to improve health care quality, and to facilitate communication or decision making showed a strong evidence for a positive effect. Overall, this review shows that interactive eHealth interventions can be effective in improving medication adherence.

### Interactive eHealth Interventions

This study showed strong evidence for a positive effect on medication adherence of eHealth interventions across various channels, including SMS, IVR, mobile apps, and calls. Our findings add robustness to the positive effect of eHealth interventions provided by previous systematic reviews and meta-analyses [46-51]. Where those authors were cautious with interpreting their findings because of low-quality studies, small sample sizes, and short follow-up, many studies we included were of high quality (22/29), had sample sizes of 100 patients or more (19/29), and follow-up of at least six months (14/29). IVR interventions that included information about health consequences suggest a stronger behavioral change, including medication-taking behavior [51]. This review confirms these findings as the included IVR interventions all contained information on the consequences of (not) taking medication as prescribed. For call, mobile app, and SMS text messaging interventions it remains unclear whether there are intervention elements (eg, content, intervention design, or extent of tailoring) that contribute to increased medication adherence because most eHealth interventions are multicomponent and elements vary widely across interventions [46,48,50].

We found a lack of convincing evidence for interventions applying an electronic monitoring device or e-training. By contrast, van Heuckelum et al [52] found a positive effect for interventions using monitoring device feedback. In our review all interventions coupled their electronic monitoring devices to the same (Way To Health) communication platform which could be a possible explanation. Yet, van Heuckelum et al [52] also included interventions that gave face-to-face feedback on adherence data collected by monitoring devices. They showed that these interventions were effective, whereas those that applied tele-feedback were not. This suggests that feedback on tele-monitoring of medication adherence is best given face-to-face.

### Intervention Strategies

To describe intervention strategies among effective interactive eHealth interventions we used Lowe’s taxonomy as it is specific for adherence interventions with clear examples for each strategy. Although other taxonomies (eg, Abraham and Michie [53], Demonceau et al [54], Kini and Ho [7]) could have been used, they show many conceptual similarities with Lowe’s taxonomy. Following Lowe’s taxonomy, we provide evidence for interactive adherence interventions aimed at teaching skills such as self-management programs, improving health care quality by coordinating medication adherence care between professionals, and facilitating communication or decision making between patient and health care professional. These results should be interpreted with caution because interventions were multifaceted and combined different strategies. Furthermore, the strategies with the highest level of evidence were also those that were less used. It is not possible to assign success to a single strategy within a multifaceted intervention. Nonetheless, the effective strategies we identified in this review suggest to be good starting points for development or selection of interventions.

### Patient Populations

Noteworthy, the included studies in our review using eHealth interventions to address medication adherence reflect 2 distinct patient populations, namely, the large patient population (eg, metabolic and cardiovascular disease) and the population where optimal medication adherence is critical (eg, immunosuppressants, antiretroviral therapy). Applying eHealth to address medication adherence can be advantageous for both populations albeit for different reasons. eHealth interventions can be accessible for large patient populations, giving health care professionals a large outreach with limited resources. For populations where optimal medication adherence is critical, eHealth interventions can be tailored to patients’ specific needs and provide continuous support.

### Study Quality

Where others found a lack of high-quality studies and stressed the importance of improving study quality [3,9,15], this review included 15 (out of 23) high-quality studies. The increase in quality presumably is a direct consequence of better reporting and study designs. We chose the Cochrane risk of bias tool (version 1) to assess study quality. This tool focusses on internal validity and does not cover all aspects of study design. We found flaws in study design that were not covered by the Cochrane risk of bias tool such as absence of sample size calculation, selection bias, and disputable (adherence) outcomes. This could have (negatively) affected the implications of the results.

### Clinical Implications and Future Research

We clustered evidence of various long-term conditions in our best evidence synthesis to provide a comprehensive overview. This overview is based on the statistically significant effects (*P*<.05) found by the authors and supported by the effect sizes we calculated. Of the 17 statistically significant (*P*<.05) interventions, 9 showed at least a small effect size (Cohen *d* ≥ 0.2) and 3 interventions showed strong odds (OR > 2.0) for effect in the intervention group.

The synthesis was limited to medication adherence and did not consider other clinical outcomes. As a result, our findings may not be applicable one-on-one to specific conditions. Next step is to study the identified effective interventions/strategies in specific long-term conditions to ascertain that this may lead to improved medication adherence and other clinical outcomes.

Only postintervention effectiveness on medication adherence was assessed in this review. Whether the found beneficial effects will be maintained over a longer period (>12 months) remains unclear. However, 12/17 effective interventions in our review had a follow-up of at least six months which is considered the shortest period to accurately assess long-term medication adherence [4].

We were surprised to find many interactive eHealth interventions that use technologies published in the 20th century. Although technology changes, applied techniques are very similar. To be able to build upon data and lessons learnt from older technologies, crosslinks between similar techniques need to be made (eg, between SMS text messaging and chat services such as WhatsApp or WeChat).

Technological developments are very fast paced and eHealth interventions continuously change. This high turnaround speed creates a need for study designs that allow continuous evaluation of interventions over a period of at least six months.

In this review intervention exposure ranged from a single call to daily messages for months. To establish a relation between exposure and medication taking behavior change, dose–response studies are called for.

### Conclusion

We found that a majority of interactive eHealth interventions are effective in improving adherence to long-term medication. Intervention strategies that improve patient’s treatment involvement and their medication management skills are most promising. While most interactive eHealth interventions were multifaceted, even simple eHealth technologies such as SMS text messaging and telephone calls can be effective in promoting medication adherence in a wide variety of patient populations.

## Appendix

Multimedia Appendix 1 MEDLINE search strategy.

Multimedia Appendix 2 Overview of the intervention strategies present in each e-Health intervention.

Multimedia Appendix 3 Sensitivity analysis.

Multimedia Appendix 4 Level of evidence of intervention strategies.

Multimedia Appendix 5 Extraction database.

## Abbreviations

AACTG: Adult AIDS Clinical Trials Group Adherence Instrument
HQ: high quality
ITAS: Immunosuppressant Therapy Adherence Instrument
IVR: interactive voice response
LQ: lower quality
MMAS: Morisky Medication Adherence Scale
OR: odds ratio
PDC: percentage of days covered
RAS: renin–angiotensin system
TIDieR: Template for Intervention Description and Replication
