# Supplementary material for: Effect of Interactive eHealth Interventions on Improving Medication Adherence in Adults With Long-Term Medication: Systematic Review
Source: J Med Internet Res. 2021 Jan 8;23(1):e18901. doi: 10.2196/18901 (PMC7822716; doi:10.2196/18901)
Supplement: Multimedia Appendix 1 [file jmir_v23i1e18901_app1.doc]

# Search terms – PubMed

**eHealth interventions**

(telemedicine[MH] | ehealth[tiab] | e-health[tiab] | electronic health[tiab] | telemedicine[tiab] | telehealth[tiab] | mhealth[tiab] | m-health[tiab] | mobile health[tiab] | emedicine[tiab] | e-medicine[tiab] | etherap*[tiab] | health technolog*[tiab] | information technolog*[tiab] | communication technolog* [tiab] | mobile tech*[tiab] | platform[TiAb] | telecare[tiab] | tele-care [tiab] |telepharmacy [tiab] | telecommunic*[tiab] | telemonitoring [tiab] |remote monitor* [tiab] | remote consult* [tiab]| telephone[MH] | phone[TiAB] | smartphone*[tiab] | mobile device*[tiab] | personal digital assistant[tiab] | pda[tiab] | wearable*[tiab] | smartwatch*[tiab] | smart-watch*[tiab] | computers[MH] | computers[TiAb] | computer[TiAb] | internet[MH] | internet[TiAb] | web[TiAb] | website [tiab] | e-mail[TiAb] | email[TiAb] | electronic mail[TiAb] |online[TiAb] | wireless[TiAb] | bluetooth[tiab] | blue tooth[tiab] | mobile applications[MH] | apps[tiab] |app[tiab]| mobile application[tiab] | interactive media[tiab] | social media[tiab] | instant messag*[tiab] | IM[tiab] | text messaging[MH] | text messag*[tiab] | SMS[tiab] | multimedia[tiab] | MMS[tiab] | chat[tiab] | social network[tiab] | teleconference*[tiab] | videoconference*[tiab] | virtual[tiab] | digital[TiAb] | (tablet*[TiAb] AND (mac | ipad | android | Microsoft | windows)))

**Medication adherence**

(medication adherence[MH] | patient compliance[MH] | medication compliance[TW] | medication non compliance[TW] | medication noncompliance[TW] | medication adherence[TW] | medication non adherence[TW] | medication nonadherence[TW] | medication adherance[TW] | medication non adherance[TW] | medication nonadherance[TW] | medication persistence[TW] | medication non persistence[TW] | medication nonpersistence[TW] | medication persistance[TW] | medication non persistance[TW] | medication nonpersistance[TW] | medicine compliance[TW] | medicine non compliance[TW] | medicine noncompliance[TW] | medicine adherence[TW] | medicine non adherence[TW] | medicine nonadherence[TW] | medicine adherance[TW] | medicine non adherance[TW] | medicine nonadherance[TW] | medicine persistence[TW] | medicine non persistence[TW] | medicine nonpersistence[TW] | medicine persistance[TW] | medicine non persistance[TW] | medicine nonpersistance[TW] | medical compliance[TW] | medical non compliance[TW] | medical noncompliance[TW] | medical adherence[TW] | medical non adherence[TW] | medical nonadherence[TW] | medical adherance[TW] | medical non adherance[TW] | medical nonadherance[TW] | medical persistence[TW] | medical non persistence[TW] | medical nonpersistence[TW] | medical persistance[TW] | medical non persistance[TW] | medical nonpersistance[TW] | drug compliance[TW] | drug non compliance[TW] | drug noncompliance[TW] | drug adherence[TW] | drug non adherence[TW] | drug nonadherence[TW] | drug adherance[TW] | drug non adherance[TW] | drug nonadherance[TW] | drug persistence[TW] | drug non persistence[TW] | drug nonpersistence[TW] | drug persistance[TW] | drug non persistance[TW] | drug nonpersistance[TW] | drugs compliance[TW] | drugs non compliance[TW] | drugs noncompliance[TW] | drugs adherence[TW] | drugs non adherence[TW] | drugs nonadherence[TW] | drugs adherance[TW] | drugs non adherance[TW] | drugs nonadherance[TW] | drugs persistence[TW] | drugs non persistence[TW] | drugs nonpersistence[TW] | drugs persistance[TW] | drugs non persistance[TW] | drugs nonpersistance[TW])

**RCT**

(randomized controlled trial [PT] | clinical trials as topic [mesh: noexp] | randomized [tiab] | randomised [tiab] | randomly [tiab] | placebo [tiab] | usual care [tiab]| trial [ti])
