# Supplementary figures and images for: Effect of Interactive eHealth Interventions on Improving Medication Adherence in Adults With Long-Term Medication: Systematic Review
Source: J Med Internet Res. 2021 Jan 8;23(1):e18901. doi: 10.2196/18901 (PMC7822716; doi:10.2196/18901)

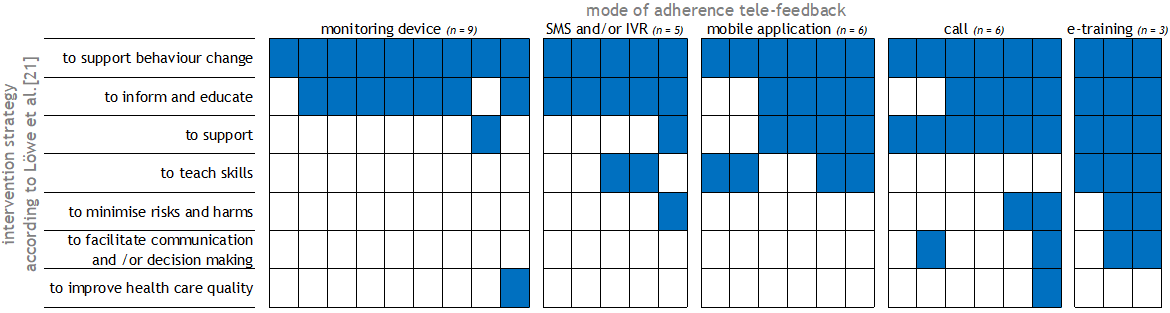

Supplement: Multimedia Appendix 2 [file jmir_v23i1e18901_app2.png]
