# Supplementary material for: Effect of Interactive eHealth Interventions on Improving Medication Adherence in Adults With Long-Term Medication: Systematic Review
Source: J Med Internet Res. 2021 Jan 8;23(1):e18901. doi: 10.2196/18901 (PMC7822716; doi:10.2196/18901)
Supplement: Multimedia Appendix 3 [file jmir_v23i1e18901_app3.doc]

**Sensitivity analysis**

| **mode of adherence tele-feedback** | **quality** | **statistically significant** | **level of evidence** |
| --- | --- | --- | --- |
| monitoring device | 9 HQ interventions | +, +, +, +, -, -, -, -, - | conflicting evidence |
|  | 0 LQ interventions |  |  |
| SMS and/or IVR | 2 HQ interventions | +, + | strong evidence for a positive effect |
|  | 3 LQ interventions | +, +, - |  |
| mobile application | 0 HQ interventions |  | conflicting evidence |
|  | 6 LQ interventions | +, +, +, +, -, - |  |
| call | 2 HQ interventions | +, - | conflicting evidence |
|  | 4 LQ interventions | +, +, +, + |  |
| e-training | 1 HQ intervention | - | moderate evidence for no effect |
|  | 2 LQ interventions | -, - |  |

*Abbreviations: SMS = short text messaging; IVR = interactive voice response; HQ = high quality; LQ = lower quality; + = p < 0.05 favouring intervention; - = p > 0.05 (no significant difference between groups). In grading the level of evidence low quality studies were disregarded when there were two or more high quality studies.*
