# Supplementary material for: Effect of Interactive eHealth Interventions on Improving Medication Adherence in Adults With Long-Term Medication: Systematic Review
Source: J Med Internet Res. 2021 Jan 8;23(1):e18901. doi: 10.2196/18901 (PMC7822716; doi:10.2196/18901)
Supplement: Multimedia Appendix 4 [file jmir_v23i1e18901_app4.doc]

**Level of evidence of intervention strategies**

| **intervention strategy** | **quality** | **statistically significant** | **level of evidence** |
| --- | --- | --- | --- |
| to support behaviour change | 22 HQ interventions | +, +, +, +, +, +, +, +, +, +, +, +, +, +, -, -, -, -, -, -, -, - | conflicting evidence |
|  | 7 LQ interventions | +, +, +, -, -, -, - |  |
| to inform and educate | 16 HQ interventions | +, +, +, +, +, +, +, +, +, -, -, -, -, -, -, - | conflicting evidence |
|  | 7 LQ interventions | +, +, +, -, -, -, - |  |
| to support | 8 HQ interventions | +, +, +, +, +, -, -, - | conflicting evidence |
|  | 7 LQ interventions | +, +, +, -, -, -, - |  |
| to teach skills | 6 HQ interventions | +, +, +, +, +, - | strong evidence for a positive effect |
|  | 3 LQ interventions | +, -, - |  |
| to minimise risk and harms | 2 HQ interventions | +, - | conflicting evidence |
|  | 3 LQ interventions | +, -, - |  |
| to facilitate communication and/or decision making | 2 HQ interventions | +, + | strong evidence for a positive effect |
|  | 2 LQ interventions | -, - |  |
| to improve health care quality | 2 HQ interventions | +, + | strong evidence for a positive effect |
|  | 0 LQ interventions |  |  |

*Abbreviations: HQ = high quality; LQ = lower quality; + = p < 0.05 favouring intervention; - = p > 0.05 (no significant difference between groups). In grading the level of evidence low quality studies were disregarded when there were two or more high quality studies.*
